# Carrying-Chair

**Published:** 1895-12-14

**Authors:** 


### EDITOR'S LETTER-BOX.

[Our correspondents are reminded that proxility is a great bar to publication, and that brevity of style and conciseness of statement greatly facilitate early insertion.]

### CARRYING-CHAIR.

Miss Laird writes: Will you give me space to correct a mistake in the little picture of "The Combined and Folding Lifting-Seat and Carrying-Chair" which accompanied your favourable notice of it in The Hospital of November 30th? The legs, which seem in that sketch to belong to the chair, are a mistake (caused, I think, by the photograph, when the legs of a table behind the chair came out rather as if belonging to it). Any legs would interfere with the very small compass into which the chair folds, and which renders it a very convenient one for travellers. As, for instance, after an invalid has been carried to and lifted into a railway carriage in the chair, it can be unhooked, folded up, and with its poles deposited in the rack till it is required again to lift the patient out.

